# Parental Smoking in the Vicinity of Children and Tobacco Control Policies in the European Region

**DOI:** 10.1371/journal.pone.0056783

**Published:** 2013-02-20

**Authors:** Viviane Kovess, Daniel J. Pilowsky, Anders Boyd, Ondine Pez, Adina Bitfoi, Mauro Carta, Ceyda Eke, Dietmar Golitz, Rowella Kuijpers, Sigita Lesinskiene, Zlatka Mihova, Roy Otten, Ezra Susser

**Affiliations:** 1 EHESP Rennes, Sorbonne Paris Cité, EA 4069 Université Paris Descartes, Paris, France; 2 Department of Epidemiology and Psychiatry, Columbia University, New York, New York, United States of America; 3 Inserm Unité Mixte de Recherche en Santé 707, EHESP, Paris, France; 4 The Romanian League for Mental Health, Bucharest, Romania; 5 Psychiatric Unit, Department of Public Health, University of Cagliari, Cagliari, Italy; 6 Yeniden Health and Education Society, Istanbul, Turkey; 7 Institute of Psychology, Leuphana University of Lüneburg, Lüneburg, Germany; 8 Behavioural Science Institute, Radboud University Nijmegen, Nijmegen, The Netherlands; 9 Clinic of Psychiatry, Faculty of Medicine, University of Vilnius, Vilnius, Lithuania; 10 Foundation for Human Relations, Sophia, Bulgaria; 11 Faculty of Social Sciences, Radboud University Nijmegen, Nijmegen, The Netherlands; 12 Mailman School of Public Health, Columbia University, New York, New York, United States of America; 13 New York State Psychiatric Institute, New York, New York, United States of America; UCL Institute of Child Health, University College London, United Kingdom

## Abstract

**Objective:**

To ascertain patterns of parental smoking in the vicinity of children in Eastern and Western Europe and their relation to Tobacco Control Scale (TCS) scores.

**Methods:**

Data on parental smoking patterns were obtained from the School Child Mental Health Europe (SCMHE), a 2010 cross-sectional survey of 5141 school children aged 6 to 11 years and their parents in six countries: Germany, Netherlands, Lithuania, Romania, Bulgaria and Turkey ranked by TCS into three level categories toward tobacco control policies.

**Results:**

A slightly higher proportion of Eastern compared to Western European mothers (42.4 vs. 35.1%) were currently smoking in but the difference was not statistically significant after adjusting for maternal age and maternal educational attainment. About a fifth (19.3%) and a tenth (10.0%) of Eastern and Western European mothers, respectively, smoked in the vicinity of their children, and the difference was significant even after adjustment for potential confounders (p<0.001). Parents with the highest educational attainment were significantly less likely to smoke in the vicinity of their children than those with the lowest attainment. After control of these covariates lax tobacco control policies, compared to intermediate policies, were associated with a 50% increase in the likelihood of maternal smoking in the vicinity of children adjusted odds ratio (AOR) = 1.52 and 1.64. Among fathers, however, the relationship with paternal smoking and TCS seems more complex since strict policy increases the risk as well AOR = 1,40. Only one country, however belongs to the strict group.

**Significance:**

Tobacco control policies seem to have influenced maternal smoking behaviors overall to a limited degree and smoking in the vicinity of children to a much greater degree. Children living in European countries with lax tobacco control policies are more likely to be exposed to second hand smoking from maternal and paternal smoking.

## Introduction

Cigarette smoking is a major determinant of health and longevity [1]. Differences in the prevalence of smoking have been described across generations, between men and women, and across regions, and related to large corresponding differences in population health and life expectancy [2]. Within the European region, a salient difference is that the current prevalence of smoking is higher in Eastern than Western countries [3].

Passive or second hand smoking (SHS) is also detrimental to health [4] and of particular concern for child health [5]. Available data suggest that exposure to parental smoking during childhood may be related to a range of child health problems, and the evidence is strong for child respiratory illnesses [6]. In numerous population studies, it has been associated with child mental health problems, particularly hyperactivity, though there is still debate as to whether these associations reflect a causal relationship [7].

Article 8 of the World Health Organization Framework Convention on Tobacco Control (2005) requires all signatory countries to adopt measures to protect people from tobacco smoke in indoor workplaces, indoor public places, public transport and other public places as appropriate [8]. Nevertheless, there was an agreement that even in countries where strict legislation is enforced, many children continue to be dangerously exposed to parental second-hand smoke [9].

Parental smoking is the most important predictor of SHS for children and children from impoverished households are more likely to be exposed [10]. Exposure to smoking at home and in cars has been associated with increased risk of children’s smoking initiation [11]. Despite an extraordinary body of literature on smoking and SHS, we know little about the frequency of child exposure to parental smoking during childhood. This crucial gap in knowledge arises in part because few population-based studies have queried parents about smoking behaviors.

To address this gap, we used data from the School Children Mental Health Evaluation project (SCHME), a multisite school-based survey of children aged 6–11 in two Western (Netherlands and Germany) and four Eastern (Romania, Bulgaria, Lithuania, and Turkey) countries in the European region in 2010 [12]. Although the main focus of the SCHME was on the mental health of European schoolchildren, the survey also included information about parental smoking habits, and in particular, about parental smoking at home in the vicinity of children. The present study compared Western and Eastern countries with respect to the prevalence of both maternal and paternal smoking in the vicinity of children. For context, we also compared the prevalence of any current maternal smoking in Western versus Eastern countries.

We also examined the relation of parental smoking patterns to tobacco control policies for countries in the European region. Tobacco control policies aim at decreasing tobacco consumption using a variety of approaches. Although these policies have been implemented in many countries, very few studies have focused on associations between these policies and parental smoking in the vicinity of children. This is important because many countries are engaged in costly campaigns to decrease smoking in the population, and parents are an important target of these campaigns.

## Methods

### Sample

The SCHME was a cross-sectional survey of schoolchildren aged 6 to 11 years and their parents in six countries: Germany, Netherlands, Lithuania, Romania, Bulgaria and Turkey. Grade schools were randomly selected in each participating country, classes were randomly selected in each school, and 6 children were randomly selected in each class. Approximately 48 children were randomly selected in each school, except in the Netherlands, where a lesser number of schools participated and therefore a greater number of children, about 120, were randomly selected in each school. To interview approximately 1000 parents, children and teachers, it was necessary to sample from 45 to 49 schools in each country. Additional information about sampling methods was included in the final SCMHE report [12]. Parents received an informational letter and a consent form to be returned to the school. If the parents did not mail the school a consent form stating their refusal to participate, the child was included.

### Participation of Children

Of the children invited to participate, 72.2% and 61.3% participated in the survey in Western and Eastern Europe, respectively. In most cases the corresponding parental respondent was the mother. Parental respondents were asked questions about smoking in the vicinity of the child in reference to both parents. Overall smoking patterns, however, were only asked in reference to the respondent. To reduce heterogeneity, we restricted these analyses to the mother’s report, and therefore, overall smoking patterns are only reported for mothers. Further information on participation, such as by country and by parental respondent, is available in the SCHME report.

### Western and Eastern Regions

The SCHME was designed to allow for broad comparisons of Western and Eastern Europe. For this purpose, SCHME classifies participants in the former West Germany and Netherlands as Western, and classifies participants in the former East Germany, Lithuania, Romania, Bulgaria and Turkey as Eastern. The division of Germany into former West and East was made because of their distinctive historical experience.

### Assessments

Data were collected from three informants: the child, the teacher and a parent. The responding parent (usually the mother) was asked to report about the other parent, regardless of marital/cohabitation status. Parental self-reports included a demographic and social questionnaire concerning household composition (including age, gender and familial link for each member), parental education (highest level completed), marital status, occupational level, rural/urban type of residence, as well as a questionnaire focusing on tobacco use. In the Netherlands the same questions were completed electronically using a secured website, though paper questionnaires were made available upon request.

The questionnaire on tobacco use included questions from a periodically administered tobacco use survey known as “Eurobarometer” [13] and from the national Canadian survey [14]. It included questions on any applicable restrictions to tobacco use for both mother and father, such as “smoking at home or in front of the child”, the main focus of this report. It also included questions on the overall smoking history of the respondent (but not the other parent), such as frequency and type of tobacco used. In this analysis, mothers who reported smoking every day or occasionally were considered current smokers.

### Tobacco Control Scale (TCS)

The “Tobacco Control Scale” (TCS) was used to measure tobacco control policies [15]. This is an inventory of European policies that was designed to examine the relation of tobacco control policies to tobacco use within and across European countries including Turkey. The TCS algorithm generates a score that quantifies the implementation of tobacco control policies at the country level. The score is based on the following six policies: (1) Price increased through higher taxes on cigarettes and other tobacco products; (2) Bans/restrictions on smoking in public and work places; (3) Extent and quality of consumer information, including public information campaigns; (4) Comprehensive bans on the advertising and promotion of all tobacco products*;* (5) Large, direct health warning labels on cigarette boxes and other tobacco products; and (6) Availability of treatment to help dependent smokers stop, including increased access to medications. Two examples will illustrate the approach used to score the TCS: (1) having implemented the recommendation to raise the cigarette price through taxation or other means is accorded 30 points: and (2) Spending on public information campaigns is accorded 15 points. Scores for each policy are added to generate an overall score. Higher overall scores, compared to lower scores, imply more restrictions. The data used by the closest available TCS survey refer to legislation in force on January 1, 2011, price data on July 1, 2010, and the budget dedicated to tobacco control in 2009. Countries were classified for this report into three groups according to their rank: high control: Turkey (61), 4 th rank; medium control: Netherlands (46) 13 th and Romania (45) 16^th^ and low control: Lithuania (41) 22 th, Bulgaria (40) 24 th and Germany (37) 26th.

### Statistical Analysis

We compared demographic characteristics of participants in Eastern and Western Europe using Chi Square analyses to estimate the significance of the differences with respect to the categorical variables listed in Table 1. Next, multivariable logistic models were used to compare (i) maternal smoking patterns in Eastern and Western Europe (Table 2) and (ii) the prevalence of maternal and paternal smoking in the vicinity of the child by region (Table 3). Adjusted prevalence was calculated as predicted marginal probabilities from the models. The models used to prepare Tables 2 and 3 were adjusted for maternal age and educational status.

**Table 1:** Sample Characteristics in Western and Eastern Europe (2010).

Demographic Characteristics	Subcategory	East Europe(% (n))	West Europe(% (n))	*p-value*
Number of children in family	Total Sample Size	(*n* = 4257)	(*n* = 884)	<0.001
	1	32.9 (1402)	10.3 (91)	
	2 or 3	52.3 (2225)	78.2 (691)	
	≥4	14.8 (630)	11.5 (102)	
Marital Status	Total Sample Size	(*n* = 3957)	(*n* = 845)	<0.001
	Single/Never Married/Separated/Divorced/widowed	16.7 (659)	9.1 (77)	
	Married/Remarried/Cohabitation/Other	83.4 (3298)	90.9 (768)	
Mother's highest level of education	Total Sample Size	(*n* = 3688)	(*n* = 778)	<0.001
	Some primary or secondary	19.4 (715)	2.2 (17)	
	Secondary completed	39.8 (1469)	30.2 (235)	
	College or technical school completed	40.8 (1504)	67.6 (526)	
Age of mother	Total Sample Size	(*n* = 4040)	(*n* = 870)	
	Years *mean (SD)*	35.7 (5.6)	40.4 (4.7)	<0.0001
	>35	52.4 (2116)	14.0 (122)	<0.001
	35–40	29.5 (1190)	34.1 (297)	
	>40	18.2 (734)	51.8 (451)	
Age of father	Total Sample Size	(*n* = 3591)	(*n* = 838)	
	Years *mean (SD)*	38.0 (5.9)	41.9 (5.1)	<0.0001
	>35	36.8 (1323)	8.5 (71)	<0.001
	35–40	33.5 (1203)	28.8 (241)	
	>40	29.7 (1065)	62.8 (526)	

West Europe: the Netherlands and West Germany.

East Europe: Bulgaria, Lithuania, Romania, Turkey, East Germany.

**Table 2:** Maternal Smoking Prevalence in Western and Eastern European Countries in 2010.

		East Europe						West Europe			*p* between East/West regions
		Bulgaria	Lithuania	Romania	Turkey	EastGermany	Total	The Netherlands	West Germany	Total	
Mother’s current smoking status	Total Sample Size	(*n* = 913)	(*n* = 971)	(*n* = 942)	(*n* = 455)	(*n* = 46)	**(** ***n*** ** = 3327)**	(*n* = 587)	(*n* = 197)	**(** ***n*** ** = 784)**	<0.001
	Daily current smoker	47.9	17.3	33.2	27.5	30.4	**31.8**	35.3	15.2	**30.2**	
	Occasional current smoker	11.4	12.6	8.8	11.0	10.9	**10.9**	3.9	7.1	**4.7**	
	Former current smoker	18.5	18.7	13.2	14.7	15.2	**16.5**	8.2	32.0	**14.2**	
	Never smoker	22.2	51.4	44.8	46.8	43.5	**40.8**	52.6	45.7	**50.9**	
% of mothers currently smoking^1^	Total Sample Size	(*n* = 856)	(*n* = 898)	(*n* = 806)	(*n* = 438)	(*n* = 45)	**(** ***n*** ** = 3043)**	(*n* = 585)	(*n* = 184)	**(** ***n*** ** = 769)**	
	Unadjusted	58.4	29.4	41.6	39.5	42.2	**42.4**	39.2	22.3	**35.1**	<0.001
	Adjusted*	57.6	30.5	39.4	33.8	37.6	**41.2**	44.8	23.2	**40.2**	0.2

* Adjusted for mother’s age as a continuous variable, and for mother’s education status; All unadjusted values are among participants without missing information on covariates.

1 Includes daily and occasional current smokers.

**Table 3:** Prevalence of Maternal and Paternal Smoking in Vicinity of Child by country and Region^1^ in 2010.

		East Europe						West Europe			*p* between East/Westregions
		Bulgaria	Lithuania	Romania	Turkey	East Germany	Total	The Netherlands	WestGermany	Total	
% of mothers smoking in vicinity of child	Total Sample Size	(*n* = 831)	(*n* = 761)	(*n* = 827)	(*n* = 465)	(*n* = 41)	**(** ***n*** ** = 2925)**	(*n* = 581)	(*n* = 182)	**(** ***n*** ** = 763)**	
	Unadjusted	32.4	10.0	20.3	16.1	19.5	**20.4**	8.8	3.9	**7.6**	<0.001
	Adjusted^2^	31.8	10.8	18.6	12.1	20.2	**19.3**	11.9	3.8	**10.0**	<0.001
% of fathers smoking in vicinity of child	Total Sample Size	(*n* = 795)	(*n* = 852)	(*n* = 782)	(*n* = 108)	(*n* = 38)	**(** ***n*** ** = 2921)**	(*n* = 579)	(*n* = 177)	**(** ***n*** ** = 756)**	
	Unadjusted	32.0	21.5	25.8	23.8	7.9	**25.7**	10.4	6.8	**9.5**	<0.001
	Adjusted^2^	31.4	22.6	24.0	18.7	10.1	**24.5**	13.5	6.5	**12.1**	<0.001

1 Western and Eastern Europe.

2 Adjusted for mother’s age as a continuous variable, and for maternal educational status. All unadjusted values are among participants without missing information on covariates.

Multivariate models were used to identify the determinants of (i) current smoking and (ii) smoking in the vicinity of the child for the mother (Table 4) and (iii) for the father (Table 5). These models were adjusted for the following potential confounders: presence of other children in the home according to age (≤3, 4–10, or 11–18 years old); parent’s age into 3 categories (≤35 years; >35 and ≤40; >40); parent’s educational level; and living with a partner versus not (single/divorced/separated/widowed). Odd ratios and 95% confidence intervals were calculated. In further analyses, we examined the relation of sociodemographic variables to smoking in the vicinity of the child, within each of the two regions.

**Table 4:** Sociodemographic Characteristics Associated with Maternal Current Smoking and Smoking in the Vicinity of the Child in Western and Eastern Europe^1^ in 2010.

		Mother smoking in vicinity of child		Mother current smoking	
		East Europe/Turkey	West Europe	East Europe/Turkey	West Europe
		OR (95% CI)	OR (95% CI)	OR (95% CI)	OR (95% CI)
Sociodemographic Characteristics	Total Sample Size	(n = 2887)	(n = 757)	(n = 2997)	(n = 762)
Presence of other children	No other children	1.00	1.00	1.00	1.00
	Children ≤3 years old	0.74 (0.56–0.98)	0.28 (0.06–1.27)	0.77 (0.62–0.96)	0.57 (0.29–1.13)
	Children 4–10 years old	0.82 (0.67–1.02)	0.64 (0.35–1.17)	0.92 (0.78–1.09)	0.59 (0.43–0.83)
	Children 11–18 years old	0.97 (0.77–1.22)	0.74 (0.37–1.46)	0.84 (0.70–1.01)	0.74 (0.51–1.08)
Mother's age	≤35 years	1.00	1.00	1.00	1.00
	>35, ≤40 years	0.84 (0.67–1.05)	0.62 (0.16–2.39)	0.76 (0.64–0.91)	0.67 (0.40–1.10)
	>40 years	0.73 (0.55–0.96)	0.30 (0.08–1.14)	0.72 (0.58–0.89)	0.94 (0.57–1.56)
Mother’s education level	None/some secondary	1.00	1.00	1.00	1.00
	Secondary completed	0.91 (0.72–1.17)	0.59 (0.15–2.32)	1.11 (0.90–1.38)	1.32 (0.45–3.89)
	College completed	0.42 (0.32–0.55)	0.25 (0.06–0.98)	0.57 (0.46–0.71)	0.84 (0.29–2.45)
Living with partner		0.57 (0.45–0.72)	0.51 (0.24–1.08)	0.57 (0.47–0.70)	0.87 (0.51–1.48)

1 Based on a multivariate logistic regression that included all variables in the table.

OR = Odds Ratio.

CI = Confidence Interval.

**Table 5:** Sociodemographic Characteristics Associated with Paternal Smoking in the Vicinity of the Child in Western and Eastern Europe^1^ in 2010.

		Father smoking in vicinity of child	
		East Europe/Turkey (*n* = 2887)	West Europe (*n* = 757)
Sociodemographic characteristics	Subcategory	OR (95% CI)	OR (95% CI)
Presence of other children	No other children	1.00	1.00
	Children ≤3 years old	0.78 (0.59–1.02)	0.83 (0.30–2.24)
	Children 4–10 years old	1.17 (0.95–1.43)	0.76 (0.45–1.28)
	Children 11–18 years old	1.15 (0.92–1.43)	1.04 (0.58–1.87)
Father's age	≤35 years	1.00	1.00
	>35, ≤40 years	0.93 (0.75–1.16)	1.01 (0.43–2.39)
	>40 years	0.81 (0.64–1.04)	0.60 (0.26–1.43)
Father’s education level	None/some secondary	1.00	*
	Secondary completed	0.95 (0.74–1.22)	*
	College completed	0.40 (0.31–0.53)	*
Living with partner		0.84 (0.61–1.15)	0.41 (0.20–0.85)

* Data not available.

1 Based on a multivariate logistic regression that included all variables in the table.

OR = Odds Ratio.

CI = Confidence Interval.

Lastly, we examined the relation of country level tobacco control policies to the following variables: mother smoking in vicinity of the child, mother currently smoking, and father smoking in vicinity of the child (Table 6). For these analyses, we used the following tobacco control policy categories: low, middle, and high, which correspond to tertiles for the TCS total score rank from 1 to 30 in this report. These models were adjusted for the variables mentioned above. All analyses were performed using STATA statistical software (v11.0, College Station, TX, USA) and significance was defined as a p-value <0.05 (two-sided significance level).

**Table 6:** Tobacco Control Policies and Parental Smoking Patterns^1^ in 2010.

	Mother currentlySmoking(n = 3759)		Mother smoking invicinity of child(n = 3644)		Father smoking invicinity of child(n = 3627)	
TCP^2^	OR(95% CI)	p	OR(95% CI)	p	OR(95% CI)	p
High	0.88(0.69–1.11)	0.3	0.82(0.60–1.11)	0.2	1.40(1.08–1.83)	0.01
Middle	1.00		1.00		1.0	
Low	1.17(1.01–1.35)	0.04	1.52(1.25–1.85)	<0.001	1.64(1.38–1.95)	<0.001

1 Based on multivariate logistic regression models for the four smoking patterns shown above. These models were adjusted for the presence of other children in the household (none/children ≤3 years/4–10 years and 11–18 years), mother’s or father’s age (≤35; >35,≤40;and.40), mothers’ educational level (none to some secondary; secondary completed; and college completed), living with partner (yes/no).

2 Tobacco control policies rank (upper third, middle third and lowest third tobacco control scores rank). Thus, low refers to the countries with the laxest tobacco control policies. Note that only one country is in the high category.

OR = Odds Ratio.

CI = Confidence Interval.

### Ethics Statement

All participating countries had the support of their governments, including their ministers of education and health and received ethical approval from the corresponding authority.

## Results

As shown in Table 1, 884 and 4257 Western and Eastern European children, respectively, participated in the survey. There were numerous statistically significant sociodemographic differences between the two regions, with smaller families and more single parents in Eastern Europe. While about two thirds of Western European mothers were college educated, only about 40% of their Eastern European counterparts had completed college. Last, both fathers and mothers were younger in Eastern Europe.

Table 2 shows the raw frequencies of maternal smoking habits, and the adjusted frequency for current smoking as defined above (daily and occasional). A slightly higher proportion of mothers were currently smoking in Eastern compared to Western Europe (42.4% vs. 35.1%; p<0.001), but after adjusting for potential confounders, such as age and for mother’s education status, the difference was minimal and non-significant (Table 2, bottom two rows).

As shown in Table 3, Eastern European mothers and fathers were significantly more likely to smoke in the vicinity of the child than their Western European counterparts. After adjusting for potential confounders, such as age and for mother’s education status, 19.3% vs. 10.0% of Eastern and Western European mothers, respectively, smoked in the vicinity of the child (p<0.001), and this was the case for 24.5% vs. 12.1% (p<0.001) of Eastern and Western European fathers, respectively.

With respect to mothers, those with a higher educational attainment were less likely to smoke in the vicinity of the child in both Western and Eastern Europe (Table 4). Among Western European mothers, college completers were much less likely to smoke in the vicinity of the child, compared to mothers with the lowest educational attainment (Odd Ratio (OR) = 0.25 95% Confidence Interval (CI) = 0.06–098). In Eastern Europe the odds of smoking in the vicinity of the child were also substantially reduced among college completers (OR = 0.42, 95% CI 0.32–0.55). As shown in Table 5, data on educational attainment were available for Eastern but not Western European fathers. Among Eastern European fathers, college completers were less likely to smoke in the vicinity of the child than fathers with the least educational attainment (OR = 0.40, 95% CI 0.31–0.53). Child’s age did not significantly add to the parental smoking model.

As shown in Table 6, mothers in countries with lax tobacco control policies (as reflected in their tobacco policy scores), compared to those in countries with intermediate policies, were more likely to smoke in the vicinity of the child (adjusted odds ratio, AOR = 1.52) and were only slightly more likely to be current smokers (AOR = 1.17). Fathers in countries with a high *or* low tobacco control policy scores were more likely to smoke in the vicinity of the child, compared to fathers in countries with intermediate policies (AOR = 1.40 for high TCP scores and 1.64 for low TCP scores). This pattern of results does not support any simple relationship between tobacco control policies and fathers’ likelihood of smoking in the vicinity of the child.

## Discussion

We have reported data on maternal and paternal smoking in the vicinity of children in Eastern and Western Europe. There were three main findings. First, Eastern European parents were about twice as likely to smoke in the vicinity of their children as their Western European counterparts. Current maternal smoking prevalence was similar, however, in Eastern and Western Europe (adjusted analysis: 41.2% and 40.2%, respectively). Thus, Western European mothers were specifically restricting their smoking in the presence of children. Second, a strong relationship was observed between parental education and smoking in the vicinity of the child, i.e. college completers in both regions were less likely to smoke in the presence of their children than those with the least education. Third, in countries with lax tobacco control policies, compared to those with intermediate policies, mothers were more likely to smoke in the vicinity of the child. The relationship of tobacco control policies to current smoking of mothers, although significant, was much weaker, suggesting that these policies may have specifically restricted maternal smoking in the vicinity of children.

The prevalence of SHS has been estimated differently for adults and children. In the case of adults it is often defined as having a spouse who smokes or being exposed to tobacco smoke at work; for children as having one or both parents who smoke [16]. Worldwide, 40% of children were estimated to be exposed to tobacco with exposure in Europe ranging from 51 to 61% with the highest exposure (61%) in Eastern Europe (including the Russian Federation) [16]. The results presented here are indicative of a lower prevalence of smoking in front of children (for mothers 20.4 and 7.6% point prevalence in Eastern and Western Europe, respectively; for fathers 25.7 and 9.5%). However, these figures are not comparable because parental smoking per se is only a very crude proxy for children’s exposure to SHS. Currently, many (in some countries most) parents who smoke choose not to do so in the vicinity of their children, so that in effect their children are not exposed to SHS from parental smoking [17]. To gauge the extent of SHS exposure in children, therefore, one has to sample children in a particular age group, and query their parents about smoking in the vicinity of their children. We were not able to identify any previous study that has done so. We found a much lower prevalence of child SHS exposure in Western, compared to Eastern European countries. There is evidence that this difference in prevalence is due to a marked decline in the proportion of children exposed to SHS in Western Europe. For example, using parents’ report and measurements of saliva nicotine in children, Sims et al. documented a 59% decline in children’s exposure to SHS in the 11 year period (1996–2006 inclusive) leading up to smoke-free legislation in England [18].

A study of five European countries (Ireland, Sweden, France, Italy and the Czech Republic) demonstrated that adult women living in Sweden and Ireland were more likely to have quit smoking in the 5 years leading to a 2008 survey than those living in the Czech Republic [19]. The investigators attributed this finding to the strong tobacco control laws implemented in Ireland and Sweden. Our findings concur with this report and add specificity to it, i.e. we found that mothers living in countries with lax tobacco control policies were more likely to smoke in the vicinity of the child than those living in countries with intermediate policies.

### Strengths and Limitations

Overall, 72.2% and 61.3% of invited children participated in Western and Eastern Europe, respectively. This level of participation is similar, however, to most other contemporary surveys. We do not know whether there was an association between participation and parental smoking status (e.g. children of smoking parents participating less than children of non-smoking parents) but it is likely that there were multiple other reasons for non-participation, e.g. privacy concerns, and lack of child interest in filling out questionnaires. The sample from East Germany was smaller than in other countries because sampling was designed for Germany as a whole, and we report separately on the prevalence of smoking in western and eastern regions of Germany. Additionally, the response rates for two important items (“current smoking” and “smoking in vicinity of child”) were particularly low in East Germany.

Although we found a relationship between tobacco control policies and smoking behaviour, laws are more likely to be enacted when supported by constituents and when they reflect societal beliefs and norms. Thus, reverse causation cannot be ruled out. Nevertheless, the association between lax tobacco control legislation and smoking in the vicinity of children is noteworthy.

The inclusion of specific information about smoking in the vicinity of children is a major strength of this study. Because mothers specifically limited smoking in the vicinity of their children, survey data on maternal current smoking patterns would not have revealed the patterns described here.

### Implications for Public Health Policy

Tobacco control measures seem to have influenced maternal smoking behavior overall to a limited degree and smoking in the vicinity of children to a greater degree, leading to a lower prevalence of SHS exposure among European children, and should be expanded and continued. Thus far, these policies do not appear to have had a similar impact on paternal smoking in the vicinity of children. Thus, fathers should be specifically targeted in anti-smoking campaigns, with an emphasis on the importance of children’s exposure to SHS. Furthermore, parents with low educational attainment should be targeted by messages appropriate to their social context, literacy and comprehension level by anti-smoking campaigns.

As suggested by Winickoff et al., pediatricians and family doctors should ask parents about their smoking patterns and specifically about smoking in the vicinity of children, and counsel parents about the impact of SHS exposure on children’s health [20]. This type of counseling requires an understanding of the need to be health-focused rather than judgmental and dismissive of smoking parents. Pediatricians and other child-focused health professionals are in the best position to provide this type of counseling because most children will have contact with the health care system, and parents expect to be counseled about matters that impact their children’s health when visiting a health professional.
